# On value compatibility: reflections on the ethical framework for pandemic healthcare distribution

**DOI:** 10.1007/s11019-025-10261-y

**Published:** 2025-03-07

**Authors:** Yijie Wang

**Affiliations:** Department of Social Science, Goethe University Frankfurt, Frankfurt am Main, Germany

**Keywords:** COVID-19, Resource distribution, Ethical framework, Value compatibility

## Abstract

An ethical framework for pandemic healthcare distribution typically encompasses multiple ethical values. However, integrating various ethical values and distributive principles into a single framework raises concerns about their compatibility and the overall coherence of the framework. This issue of value compatibility could lead to moral inconsistencies within the ethical framework, leading to practical indetermination when facing conflicting implications. This paper offers a methodological resolution to the compatibility problem, serving as an effective tool to mitigate the impact of value conflicts where possible. It proposes four pathways: specifying values rather than balancing them, incorporating values rather than weighing them, reinforcing values rather than aggregating them, and seeking scientific evidence. By developing coherent ethical frameworks where values do not contradict each other, this approach also enhances practical ethical decision-making. Using the COVID-19 vaccine distribution as a case study, this approach demonstrates how conflicting values can yield practical prioritization strategies, such as allocating vaccines to healthcare and essential workers, addressing multiple layers of disadvantage, and assessing age-related prioritization. Reflecting on the compatibility of values within ethical frameworks offers crucial insights beyond COVID-19, contributing to the development of robust ethical frameworks for future public health crises.

Since the onset of the COVID-19 pandemic in early 2020, the ethical distribution of scarce medical resources has become a central topic. To distribute these scarce medical resources ethically and efficiently, numerous national and global frameworks were proposed and implemented (National Academies of Sciences, Engineering, and Medicine et al. 2020; Dawson et al. 2020; DIVI Deutschen Interdisziplinären Vereinigung für Intensiv- und Notfallmedizin 2020; DOH Department of Health Ireland 2020; Emanuel et al. 2020a, b; Feiring et al. 2020; Nuffield Council on Bioethics 2020; Swiss Academy of Medical Sciences 2020; Toner et al. 2020; World Health Organization 2020; see also Jöbges et al. 2020; Joebges and Biller-Andorno 2020; Aquino et al. 2021; Lawrence et al. 2022). Despite the timely guidance for healthcare practice, these frameworks may neglect one significant ethical problem, which I identify as the compatibility problem. The compatibility problem questions whether multiple ethical values and distributive principles are compatible with each other and can constitute a coherent ethical framework for pandemic healthcare resource distribution. This paper aims to highlight and address this problem with a methodological approach, serving as an effective tool for mitigating the impact of value conflicts where possible. While COVID-19 serves as the context illustrating the complexities of ethical decision-making during public health emergencies, the analysis extends beyond this specific context. It will provide new insights beyond the COVID-19, contributing to enhancing preparedness for future public health emergencies.

## The compatibility problem

An ethical framework for the distribution of pandemic healthcare resources typically encompasses multiple ethical values, such as the utilitarian value of benefiting people and limiting harm, the egalitarian value of equal moral concern, the prioritarian value of prioritizing the worse off, equity and background justice, helping people in need, reciprocity, and narrow social utility. The multi-value framework is justified because no single ethical value or distributive principle is sufficient on its own to capture all morally relevant considerations in the complex healthcare rationing during public health emergencies (Cookson and Dolan 2000; Persad et al. 2009; Gupta and Morain 2021). Moreover, it can avoid categorical exclusion of treatment to certain groups and extend it to a larger group of people than a single-value allocation, when relevant moral considerations are properly identified and justified and they are complementary to each other (White et al. 2009).

However, when combining multiple ethical values and distributive principles into one framework, we encounter greater complexities, redundancies, and controversies (Arras 2005; Persad et al. 2009; Scheunemann and White 2011; Emanuel et al. 2020a, b; Toner et al. 2020; Wasserman et al. 2020; Williams and Dawson 2020; Raus et al. 2021; Rhodes 2021; Aquino et al. 2021; Goozen 2021; Saxena et al. 2021; Curran and John 2022; März et al. 2022). As Williams and Dawson point out, “There was a tendency in some of the literature for authors to list principles or values for consideration in vaccine rationing, without necessarily explaining… whether the listed values were complementary or even reconcilable with each other (J. H. Williams and Dawson 2020, p. 4)”. The “incompatibility (Rhodes 2021, p. 624)”, “internal inconsistencies (Aquino et al. 2021, p. 186)”, “conflicts (ibid.)”, “deep moral tensions between efficiency, equity, and responding to those facing death (Scheunemann and White 2011, p. 1629)” raise significant moral and practical challenges that should be taken seriously.

I call the problem of whether multiple ethical values and distributive principles are compatible with each other and can constitute a coherent ethical framework for pandemic healthcare resource distribution, in short, *the compatibility problem*. This problem is prevalent in the multi-value framework. For example, most frameworks have a utilitarian focus, aiming to maximize relevant benefits and minimize harm during the pandemic. However, choosing different utilities may not be compatible with each other and can lead to different resource allocation schemas. The idea of saving the most lives generally emphasizes preventing immediate deaths, often prioritizing individuals, like the elderly, based on the severity of illness and their survival prognosis. In contrast, saving the most life years tends to favor younger individuals who have more potential years of life remaining.^1^ Meanwhile, saving the most quality-adjusted life years (QALYs) takes into account both age and comorbidities, giving priority to those who are likely to live longer and healthier lives. Incompatibilities also arise between values such as utilitarianism and egalitarianism. The egalitarian approach advocates for distributing scarce medical resources equally among all individuals, prioritizing fairness over maximizing overall population health. This approach critiques the utilitarian focus on outcomes, which aggregates benefits across the population, arguing that it fails to respect equal worth of life. These conflicting values illustrate the fundamental divide between consequentialist and deontological ethical theories. Consequentialism determines moral rightness based on the outcomes of actions, leading to practices like prioritizing younger individuals to maximize life years, which may seem to discriminate against the elderly. In contrast, deontological ethics insists that the morality of an action depends on the action itself, emphasizing equal moral consideration for all lives grounded in the inherent worth and dignity of individuals, regardless of potential outcomes.^2^ In view of these fundamental divergences, it is questionable whether multiple ethical values and distributive principles are compatible with each other and can constitute a coherent ethical framework for pandemic healthcare resource distribution.

The prevalent compatibility problem exerts a profound impact. On one hand, it engenders moral inconsistency, which undermines the integrity of ethical frameworks. Consistency is crucial in ethics, demanding that our moral standards, values, and actions be rational and non-contradictory. The numerous conflicts within and between ethical values and distributive principles significantly challenge the principle of consistency in ethical frameworks. When faced with an ethical framework plagued by moral inconsistencies, doubts naturally arise regarding the necessity of constructing such a framework. This uncertainty further undermines the idea of a shared ethics grounding healthcare.

On the other hand, it leads to practical indetermination. The numerous incompatibilities within and between ethical values and distributive principles create a web of conflicting implications, leading to a state of indetermination when implementing them in practice. The practical contradictions and indetermination deepen the gap between theory and practice, presenting severe challenges in translating ethical values and distributive principles into operational guidance. If such contradictions and indetermination persist, the foundation of a shared ethical grounding for healthcare would be undermined, leading to arbitrary and irrational decision-making. Given these profound impacts, it becomes imperative to address the compatibility problem.

Before proceeding to the next section, it is necessary to make two preliminary remarks to clarify potential misunderstandings.^3^ First, this paper examines the compatibility problem and its resolution primarily in a narrow, technical sense, focusing on ensuring internal consistency—where the ethical values and distributive principles within a framework do not contradict one another. However, compatibility problems must also be situated within a broader ethical deliberation context alongside other considerations, such as fairness, feasibility, justifiability, inclusivity, and the societal values that underpin ethical frameworks. While these broader considerations may be implicitly integrated into the process of achieving internal consistency, they also constitute a more expansive compatibility problem and resolution beyond the scope of this paper. With this narrow and moderate focus, the compatibility resolution proposed here serves as a necessary but not sufficient condition in determining the overall appropriateness of an ethical framework.

Second, in identifying and addressing the compatibility problem, I do not intend to undermine the significance of moral pluralism or moral disagreement, nor do I suggest that all value conflicts are resolvable at a principled level. As many scholars have argued, some degree of moral tension is inevitable and even desirable in pluralistic societies, as it reflects diverse perspectives and respects agents’ autonomy (Berlin 1969; Rawls 1996; Chang 1997). Furthermore, there are genuinely irresolvable conflicts between values that may resist reconciliation (Williams 1981; Larmore 1987). This impossibility is evident in public health emergencies such as the COVID-19 pandemic, where tragic triage decisions—such as determining who lives and dies—highlight the gravity of such conflicts (Beall 2020).

While accepting these premises, I contend that such conflicts are not (or should not be) necessarily irresolvable on a practical level. In real-world contexts, radical pluralism can be reframed into a workable pluralism, where multiple values can attain a degree of compatibility sufficient to offer practical guidance. Compatibility resolution thus does not aim to eliminate moral conflicts but to mitigate the impacts of these conflicts, providing a framework for making defensible decisions under pressure and prioritizing actionable guidance. This practical approach to value compatibility emphasizes the importance of addressing these conflicts pragmatically. While such decisions may remain morally imperfect, they succeed in addressing urgent practical needs, thereby enabling better preparation for future emergencies.

## A methodological approach to the compatibility problem

While many scholars acknowledged the importance of the compatibility problem, they admitted that “The issue of how to combine different principles to arrive at a morally sound and easy-to-use approach is …… difficult to resolve (Goozen 2021, p. 32)”. In the context of allocating healthcare resources during the COVID-19 pandemic, scholars have addressed the compatibility problem by seeking trade-offs between conflicting values. They proposed methods such as point systems or weighted lotteries to assign varying degrees of moral weights to these values (Liu et al. 2020; Jansen and Wall 2021; White et al. 2022). However, this substantive approach encounters problems of precision and arbitrariness. It seems arbitrary, for example, to assign a 25% increase in chances to people who come from disadvantaged communities in the weighted lottery proposed by White et al. (2022). Why not 30%, or 80%? More fundamentally, it seems impossible to specify or justify a covering value, according to which different values can be reduced to a common measure and be commensurable. Consequently, the effectiveness of the trade-off approach in guiding complex ethical decision-making, where multiple competing values must be reconciled, is compromised.^4^

Instead of the trade-off approach, I propose a methodological approach with four reasonable pathways, serving as an effective tool for mitigating the impact of value conflicts on a practical level. They are: (1) specifying values rather than balancing them, (2) incorporating values rather than weighing them, (3) reinforcing values rather than aggregating them, and (4) seeking scientific evidence. The polarization of methods serves a strategic purpose. This analysis aims to underscore the limitations of default methods such as balancing, weighing, and aggregating values, while emphasizing the necessity of alternative approaches, including specifying, incorporating, and reinforcing values supported by scientific evidence. Here, I do not intend to dismiss default methods entirely but to recognize their continued relevance when appropriately refined. Therefore, I propose viewing these alternative pathways as complementary strategies that address the shortcomings of default approaches.

### Specifying rather than balancing

The first pathway emphasizes the advantage of the method of specification in addressing compatibility issues. The methods of specification and balancing are generally viewed as distinct and functioning differently (Beauchamp and Childress 2013). While specification is “a process of reducing the indeterminacy of abstract norms (ibid., 17)” and is “especially useful for developing more specific policies from already accepted general norms (ibid., 20)”, balancing is “concerned with the relative weights and strengths of different moral norms (ibid.)” and is “particularly well suited for reaching judgments in particular cases (ibid.)”. This dichotomy, however, is misleading. Specification is particularly well-suited to addressing the compatibility problem and can complement traditional balancing methods.

First, conflicting norms can be specified in such a way that the need for balancing them is avoided. Consider the value of equal moral concern in the ethical framework for COVID-19 vaccine distribution, among the various definitions and interpretations, such as equality of expenditure per capita, equality of inputs per capita, equality of input for equal need, equality of access for equal need, equality of utilization for equal need, equality of marginal met need, equality of health (Culyer and Wagstaff 1993), some are compatible with other ethical values while others are not. For example, equality of health, the *ex post* end-state equal health status, cannot be compatible with the value of prioritizing the worse off. Some worse-off health conditions, such as unrecoverable diseases, severe physical disabilities, or the genetic disorder of Down syndrome, cannot be raised to the same level of health as others under current medical conditions. This is, however, not the case with equality of opportunity to healthcare. Equality of opportunity to healthcare requires treating everyone equally and not discriminating on the basis of morally irrelevant differences such as sex, race, and religion. It implies that people with different healthcare needs should be treated differently and is thus compatible with the value of prioritizing the worse off.^5^ If we carefully tailor the value of equal moral concern into equality of opportunity to healthcare, the value of equal moral concern is compatible with the value of prioritizing the worse off, and the need to balance them would not even come to the stage.

Second, specification is clear and action-guiding. Unlike balancing, which relies on the moral agent’s judgment on the quantitative weighting or discounting “through practical astuteness, discriminating intelligence, and sympathetic responsiveness (Beauchamp and Childress 2013, p. 22)”, specification emphasizes the significance of morally and publicly justifying how we adapt moral norms qualitatively to individual situations. As Richardson suggests, “the model of specification concurs with the balancing approaches in seeing a need to qualify our commitments, but insists that this be done not by a quantitative weighting or discounting but instead by qualitatively tailoring our norms to cases (Richardson 1990, p. 283)”. The qualitative specification has four essential modes: specification itself, extensional narrowing and glossing, the components of specification, and sharpening (Richardson 2000). Specification itself involves a direct and explicit articulation of ethical principles concerning a particular situation. Extensional narrowing and glossing focus on tailoring moral norms to particular cases while preserving their underlying essence. The components of specification involve breaking down complex ethical values into their constituent elements. Sharpening serves as a prerequisite for successful specification. These modes offer a clear and action-guiding way of specifying ethical values with respect to other co-existing values in an ethical framework.

In sum, specification demonstrates significant advantages in resolving the compatibility problem, particularly in clarifying and specifying ethical principles in a more action-guiding manner. Therefore, specification can serve as a valuable refinement of the default method of balancing to enhance the precision and applicability of ethical values.

### Incorporating rather than weighing

The second pathway highlights the advantages of incorporating different values and principles as a way to enhance ethical coherence, offering a complementary approach to assigning weights. Weighing the moral weights of different ethical values and distributive principles has two problems. First, it wrongly presupposes that different values and principles are commensurable with each other given that no covering value exists or can be justified. For values to be commensurable, there should be a covering value according to which different values can be reduced to a common measure (Chang 2002). However, it is questionable whether such a covering value exists or can be justified. Second, following value incommensurability, it is questionable how the weighing method could justify quantitative relationships between different values.

In contrast, incorporation aims to incorporate and unite different values and principles. It can be achieved by an overarching principle. For example, Rawls’ difference principle establishes a *pro tanto* relationship between equality and priority: inequalities in the distribution of goods are permissible only if those inequalities are to the greatest benefit of the worst-off members of society (Rawls 1999). By doing so, the difference principle unites the values of equality and priority, recognizing that both are essential in shaping a just society. Another example is the “protean research-limiting principle”, which incorporates bioethical principles of autonomy, beneficence/non-maleficence, and justice. The principle is stated as follows: “It is impermissible to engage in research on human subjects unless the principles of autonomy, beneficence, and justice are adequately satisfied (Richardson 2000, p. 301)”. By doing so, the four principles are not weighed against one another. Instead, they are united into one overarching principle which recognizes the value of each in the research context.^6^

Following this line of reasoning, resolving the compatibility problem can be enhanced by incorporating different ethical values and distributive principles. Rather than viewing incorporation as a strict alternative to weighing, it can serve as a complementary approach that promotes a more inclusive and comprehensive ethical framework by integrating diverse moral considerations without relying solely on trade-offs.

### Reinforcing rather than aggregating

The third pathway to the compatibility problem is the moral reasoning of reinforcement, which has a distinct advantage over mere aggregation. Simply aggregating relevant benefits and harms fails to respect equal moral worth by allowing the welfare of a few to be sacrificed in favor of the general. It is indeterminant when two cases have the same aggregative value but differ qualitatively.

Unlike aggregation, reinforcement brings the crucial qualitative dimension back into focus. It has three key elements. The first is the reinforcement of moral arguments. When multiple ethical values align and support prioritizing a particular group, that group acquires special moral importance because multiple arguments reinforce its moral force. A typical example is the prioritization of vaccines to healthcare and essential workers during a pandemic, which is justified by the value of benefiting people and limiting harm, narrow social utility, and reciprocity. Firstly, prioritizing this group prevents deaths, reduces transmission, and maintains socioeconomic stability by acknowledging their critical role in healthcare systems and public safety during high-risk situations. Secondly, the principle of narrow social utility underscores the significant impact healthcare workers have in saving lives, which is magnified during a pandemic. Lastly, the principle of reciprocity recognizes the sacrifices these workers make by facing considerable risks, emphasizing a societal obligation to prioritize their well-being not just for potential outcomes, but for their substantial contributions during the pandemic. When such reinforcement happens, the normative moral force of prioritizing healthcare and essential workers becomes more compelling.

The second is the reinforcement of the multiplier effect, which is crucial when prioritizing certain key individuals to amplify overall benefits. A typical example is that during World War II, the scarce medical resource of penicillin was prioritized for soldiers who had venereal disease rather than those suffering from battle wounds to achieve the multiplier effect of increasing the manpower at the front (Beecher 1970). Similarly, in the context of a pandemic, vaccines should be prioritized for frontline healthcare workers to achieve the multiplier effect of saving more lives, given that healthcare workers can ultimately save more lives through their work. As these cases show, reinforcement introduces a qualitative dimension to healthcare decision-making, focusing on the amplification of benefits rather than mere aggregation.

The third is the reinforcement of disadvantages. Disadvantages tend to cluster, creating compounded challenges for vulnerable populations (Wolff and de-Shalit 2007, 2021). In the context of the COVID-19 pandemic, vulnerable populations often suffer from a combination of worse health conditions, worse social conditions, and limited access to healthcare resources (Bambra et al. 2020). Such cluster of disadvantages indicates the presence of structural injustices that systematically lead to intersectionality and vicious spirals for certain groups (Bowleg 2020; Lewicki 2021). As a response, de-clustering disadvantages and prioritizing the multiply disadvantaged should be a paramount strategy. This is not only morally imperative for deontological reasons but also practically efficient for consequentialist reasons. Instead of merely focusing on minimizing aggregative harms, de-clustering disadvantages has the potential to break down the reinforcement of various worse-off conditions, which is the crux of reducing harm.

In sum, reinforcing provides a valuable refinement to aggregating in resolving the compatibility problem. The reinforcement of moral argument, multiplier effect, and de-clustering disadvantages brings the crucial qualitative dimension back into focus. By adopting the moral reasoning of reinforcing, a more reasonable and practical ethical framework could be established.

### Seeking scientific evidence

In addition to the previously discussed pathways, scientific evidence plays a crucial role in addressing the compatibility problem within ethical frameworks. It is important to clarify that scientific evidence does not dictate ethical decisions. It cannot determine which ethical choices are correct or which aim a society should value most during public health emergencies—they belong to the domain of ethics. Instead, scientific evidence serves as a crucial tool in facilitating science-informed ethical decision-making, enhancing the applicability and relevance of abstract ethical values and principles in a dynamic world. Specifically, empirical evidence functions in three critical capacities: as a touchstone for verifying the practical implications of ethical theories, as a mediator in reconciling conflicting ethical values, and as a tiebreaker in making decisions when ethical principles are in equipoise. Each of these roles underlies the indispensable support that scientific data provides in refining and applying ethical considerations to real-world scenarios.

Firstly, scientific evidence is a touchstone for verifying the practical implications of ethical theories. It can call into question the validity of morally justified values and principles in a specific context. For example, during COVID-19, though ethically justified, it is questionable to prioritize the young based on fair innings or life-cycle argument because they have a far lower fatality rate than the elderly. Ignoring the scientific evidence would lead to a dogmatic universalism of ethical values and distributive principles. Instead, when conflicts occur, ethical values should remain open to reevaluation and adaptation based on the best available evidence to ensure their relevance and effectiveness in an ever-changing world.

Secondly, scientific evidence is a mediator in reconciling conflicting ethical values. Taking the utilitarian value of saving the most lives and the most life years for example, these two principles often come into conflict when it comes to age priority. If we aim to save the most lives, vaccine priority should be given to the elderly who face significant risk of mortality when infected. In contrast, if we aim to save the most life years, vaccine priority should otherwise be given to the young who have more remaining life years to live. This complication, however, could be alleviated when we reexamine the scientific evidence. According to a simulation which takes into account the COVID-19 infection fatality rate (the young 0.1%, whereas the elderly 11.6%), the vaccine efficacy (90%), the population characteristic (people aged 20 years comprise 85% of the population, whereas people aged 73 years comprise the remaining 15% of the population), the objective of saving the most life years would also prioritize the elderly over younger people because it would save 63.7 more life years (Jecker et al. 2021). This fact suggests that certain scientific evidence could serve as a mediator to reconcile conflicting ethical values and distributive principles, which helps to alleviate the compatibility problem.

Thirdly, scientific evidence is a tiebreaker in making decisions when ethical principles are in equipoise. Taking again age priority for example, while different interpretations of egalitarian and utilitarian values can justify the prioritization of both the young and the elderly, scientific evidence can offer guidance depending on changing circumstances. An empirical study demonstrates that when there are stark differences in mortality risk with age or when the reproductive number R_0_ is high, older people should be prioritized for vaccination; conversely, when there are high vaccine efficacy, high social contact rates among younger people, and widespread adherence to nonpharmaceutical interventions, priority should be shifted towards the young (Fitzpatrick and Galvani 2021). This adaptability highlights the indispensable role of scientific evidence in resolving the compatibility problem by providing an objective and empirical basis for decision-making in dynamic circumstances.

## Policy suggestions: COVID-19 vaccine distribution as a case study

The methodological approach to the compatibility problem seeks to develop coherent ethical frameworks where values do not contradict each other, thereby enhancing practical ethical decision-making. In the context of COVID-19 vaccine distribution, the compatibility resolution offers three illustrative policy considerations: (1) prioritize healthcare and essential workers COVID-19 vaccines, acknowledging their indispensable roles during the pandemic that are supported by a convergence of values; (2) prioritize the multiply disadvantaged, recognizing the compounded vulnerabilities exacerbated by the pandemic and thereby incorporating both consequentialist and deontological arguments; (3) ensure that age prioritization in the distribution of vaccines is informed by robust scientific evidence. While the distribution of COVID-19 vaccines provides a valuable case study for applying this approach, the insights derived extend beyond the pandemic to broader public health preparedness. By fostering ethical coherence and mitigating value conflicts, this framework contributes to the development of adaptable, principled strategies for resource allocation in times of crisis, enhancing both ethical integrity and practical effectiveness in future preparedness efforts.

### Prioritizing healthcare and essential workers

When examining the conflicting values and principles in an ethical framework of COVID-19 vaccine allocation, it is surprising that there is a tremendous consensus in prioritizing healthcare and essential workers. Healthcare workers usually include physicians, nurses, emergency medical personnel, dental professionals and students, medical and nursing students, laboratory technicians, pharmacists, hospital volunteers, and administrative staff. Essential workers generally include military personnel, police and fire personnel, first responders, communication services, transporters, food and sanitary workers, teachers, and other school workers. They acquire special moral importance because doing so is supported by multiple ethical values, including the value of benefiting people and limiting harm, narrow social utility, and reciprocity. These aligned values not only justify but reinforce the normative moral force behind the prioritization of this critical workforce, reflecting a robust ethical endorsement of their key role during the pandemic.

First, the value of benefiting people and limiting harm aims to prevent deaths, reduce transmission rates, save lives and life years, and promote socioeconomic well-being. During the pandemic, it is evident that frontline healthcare and essential workers are indispensable in maintaining healthcare systems and public safety. At the same time, they are at high risk of exposure to the virus and high risk of infection, transmission, morbidity, and mortality due to frequent contact with COVID-19 patients, high public contact jobs, and high-density workplaces. Prioritizing healthcare and essential workers can avoid absenteeism, reduce transmission, prevent deaths, and protect healthcare systems and public safety. As a modelling study shows, in such a setting with a population of 5 million, vaccinating essential workers sooner prevents over 200,000 infections, and over 600 deaths and produces a net monetary benefit of over $500 million (Mulberry et al. 2021).

Second, in line with the utilitarian rationale, narrow social utility is also widely recognized and applied in the context of a pandemic. In contrast with broad social utility, it considers the comparative social value of an individual in achieving the specific goal as a relevant and even decisive consideration under some circumstances, if and only if his or her contribution is indispensable for achieving that specific goal. During the pandemic, healthcare and essential workers perform their narrow social utility in promoting public and individual well-being and preventing the dreadful consequences owing to their profession. In other words, they are instrumentally valuable to ending the crisis. Besides, prioritizing healthcare and essential workers during a pandemic has the multiplier effect of saving more lives and maintaining social functioning owing to their critical medical expertise and social roles.

Third, besides the utilitarian value, deontological reasons also support prioritizing healthcare and essential workers. This prioritization does not rely on the consequences, but rather on the rightness of the act itself. The value of reciprocity emphasizes a society’s duty to reward those who have benefited society while bearing significant risks. It prioritizes healthcare and essential workers not for the consequences that might be generated but simply because healthcare and essential workers contributed to ending the pandemic while bearing significant risks. Thus, based on their contribution and risks, these workers possess a strong moral claim to be rewarded and protected on reciprocal terms.

Prioritizing healthcare and essential workers corresponds to the four pathways and is thus compatible with most ethical values. First, this prioritization is specifically tailored to a group that acquires broad ethical support, embodying a targeted approach where the various ethical values converge rather than conflict. Second, it incorporates and reflects these values rather than weighing them against each other, reflecting a unified ethical stance. Third, this approach is supported by multiple values including both consequentialist and deontological arguments, which reinforces its normative moral force. Besides, this prioritization generates a multiplier effect, contributing to the qualitative improvement of life-saving efforts in a way that goes beyond mere numerical aggregation. Fourth, empirical support from scientific modelling studies further validates that this prioritization benefits people and limits harm, offering robust evidence of its ethical and practical efficacy. Consequently, the prioritization of healthcare and essential workers not only adheres to the proposed pathways but also strengthens the ethical framework’s consistency and practical applicability.

### Prioritizing the multiply disadvantaged

A second policy suggestion followed by the four pathways is prioritizing the multiply disadvantaged. This prioritization recognizes that while the pandemic highlights the vulnerability of all human beings in the face of the virus, certain groups experience compounded vulnerabilities due to a confluence of health and socioeconomic disadvantages. Health disadvantages could include a high risk of severe disease and death, high infection risk, ageing, living with comorbidities, living in high-density housing, and working in a highly contagious environment. Socioeconomic disadvantages often intersect with these health issues and could include low income, poverty, loss of home ownership, risk of unemployment, little access to healthy food, having no insurance coverage, and residency in deprived areas. Furthermore, social characteristics such as belonging to marginalized groups including migrants, undocumented populations, racial, ethnic, and religious minorities, as well as those with disabilities or those involved in sex work, often exacerbate these vulnerabilities.

While it is essential to address background inequalities and injustices, the direct prioritization of the multiply disadvantaged in COVID-19 vaccine allocation emerges as a crucial measure. This principle incorporates multiple ethical values, including benefiting people and limiting harm, prioritizing the worse-off, and helping people in need. First, the value of benefiting people and limiting harm aims to promote health and socioeconomic benefits and prevent health and socioeconomic harms. Those who are multiply disadvantaged are usually physically vulnerable to COVID-19 (for example, those who are living in high-density housing) while suffering from socioeconomic disadvantages (for example, those who have low income to afford better housing). Therefore, the value of benefiting people and limiting harm would support prioritizing this group to prevent harm. Moreover, given that disadvantages tend to cluster, prioritizing this group of people has the potential to break down the reinforcement of various worse-off conditions, which is the crux of reducing harm.

Second, the value of prioritizing the worse off uses deontological reasons to justify this prioritization: it is intrinsically valuable to benefit the worse off than the better off. This prioritization does not follow the rationale of reducing marginal utility (utilitarianism) or reducing inequalities (egalitarianism). The worse-off conditions are identified based on their absolute, not relative, level of well-being. It supports prioritizing the multiply disadvantaged because they suffer a cluster of disadvantaged conditions and should be prioritized intrinsically.

Third, helping people in need is fundamental in social justice, healthcare resource allocation, and the context of COVID-19. It aims to allocate healthcare resources to those who have objective-given needs based on scientific evidence. It supports prioritizing the multiply disadvantaged because they have medical needs based on health and socioeconomic evidence.

Prioritizing the multiply disadvantaged follows the four pathways and is compatible with most ethical values. First, this approach targets a specific group, rendering the need to balance conflicting ethical values largely irrelevant. Second, it serves as an overarching principle that incorporates multiple ethical perspectives, including both consequentialist and deontological arguments. Given that disadvantages tend to cluster and reinforce one another, prioritizing the multiply disadvantaged is not only morally imperative for deontological reasons but also practically efficient for consequentialist reasons. Third, this strategy reflects deep moral reasoning related to reinforcement. Rather than simply aiming to minimize aggregate harms, prioritizing the multiply disadvantaged has the potential to addresses the root causes of these harms by disrupting the cycles that exacerbate conditions for the worst-off, thereby effectively reducing overall harm. Fourth, the identification of the multiply disadvantaged is underpinned by robust scientific evidence, suggesting the feasibility of creating a comprehensive COVID-19 disadvantage index. This index would systematically capture health and socioeconomic disadvantages intensified during the COVID-19 pandemic, providing a grounded and empirical basis for targeted interventions.

### Age prioritization

A third vital policy suggestion followed by the resolution of the compatibility problem is age prioritization. Unlike the consensus on prioritizing healthcare and essential workers, vaccine distribution by age is controversial. Ethical perspectives vary significantly across different age groups, often leading to divergent implications even within the same value. For example, the utilitarian value of saving the most lives emphasizes preventing immediate deaths, often prioritizing individuals like the elderly, while another utilitarian stance of saving the most life years tends to favor younger individuals who have more potential years of life remaining. Similarly, although egalitarian values typically oppose age discrimination, advocating for the equal value of all lives, arguments based on fair innings or life stages could support prioritizing the young. This reflects the egalitarian ideal that everyone should have an equal chance to experience a normal lifespan or through stages of life.

The debate over age prioritization in COVID-19 vaccine allocation can be informed by scientific evidence, which serves several critical roles in ethical decision-making. Initially, scientific evidence acts as a touchstone for assessing the applicability of ethical values and distributive principles within specific contexts. A fundamental scientific observation is that younger individuals exhibit significantly lower COVID-19 fatality rates compared to older adults. Consequently, prioritizing the young based solely on the fair innings or life-cycle arguments appears not applicable in this context, despite their ethical validity.

Furthermore, scientific evidence can serve as a mediator to reconcile conflicting ethical values and distributive principles, thereby alleviating the compatibility problem. In scenarios where age-based priority is debated, the conflict between maximizing the number of lives saved and the number of life years saved can be reconciled through careful examination of relevant data. According to a simulation, the objective of saving the most life years also prioritizes the elderly over younger people because it would save 63.7 more life years (Jecker et al. 2021).

Lastly, scientific evidence can be a tiebreaker under varying conditions. Research indicates that in situations with stark differences in mortality risk with age or when the reproductive number R_0_ is high, older people should be prioritized for vaccination; conversely, when there are high vaccine efficacy, high social contact rates among younger people, and widespread adherence to nonpharmaceutical interventions, priority should be shifted towards the young (Fitzpatrick and Galvani 2021). Informed by scientific evidence, the framework could be more adaptive to navigate complex ethical decisions in pandemic response.

As shown above, the resolution of the compatibility problem in age prioritization primarily relies on scientific evidence. This reliance does not diminish the role of ethics; rather, it underscores it. Scientific evidence comes into play as a supportive tool when ethical dilemmas arise. In such cases, it provides objective and empirical insights that inform and refine ethical decision-making. Thus, while scientific evidence guides the application of conflicting ethical principles, it does not supplant the foundational role of ethical judgment.

## Conclusion

The issue of value compatibility extends beyond normative ethics; it critically impacts the moral consistency and practical applicability of ethical frameworks for pandemic healthcare rationing. By adopting the proposed methodological approach, the framework could empower various ethical values to operate coherently while yielding practical guidance. In such a framework, multiple conflicting values are not solely balanced, weighed, and measured in an aggregative manner. Rather, they are specified into concrete groups, allowing for the incorporation of diverse ethical reasonings, reinforcing both the moral force and effectiveness of the decisions, and supported by robust scientific evidence. In the context of COVID-19 vaccine distribution, the compatibility resolution demonstrates how conflicting values can yield practical prioritization strategies. It offers three illustrative policy suggestions, including allocating vaccines to healthcare and essential workers, addressing multiple layers of disadvantage, and assessing age-related prioritization.

While the COVID-19 vaccine distribution serves as a case study, the insights gained from this approach—centered on specifying, incorporating, and reinforcing values supported by scientific evidence—has broader relevance for enhancing preparedness for future public health crises. The compatibility problems addressed in this paper are not unique to the context of COVID-19 or vaccine distribution but are likely to emerge in a wide range of resource allocation scenarios in future emergencies. This methodological approach can be adapted to guide ethical decisions for ICU bed prioritization, ventilator allocation, or the equitable distribution of protective equipment, where conflicting norms and values also surface. While implementation details will necessarily vary depending on the nature of the resource and the context, the four pathways remain consistent and transferable, offering a robust foundation for ethically sound and actionable decision-making. By integrating this approach into planning and preparedness efforts, ethical frameworks can be strengthened to ensure more coherent and effective responses to future public health challenges.

## Data Availability

The author confirms that no consent is required.

## Footnotes

1 The discussion thus far has focused on general scenarios; however, some might argue that in the specific context of COVID-19, the objectives of saving the most lives and life years are not inherently conflicting and both naturally prioritize the elderly (Jecker et al. 2021). This alignment underscores the pivotal role of scientific evidence in mediating ethical decisions. I will discuss it in greater detail in the next section.

2 Further incompatibilities may arise when each principle is interpreted either *ex ante* or *ex post*. See Curran and John (2022) for a detailed illustration of this point.

3 I thank two anonymous reviewers for pointing out these two concerns.

4 Other approaches to resolving the compatibility problem in the context of COVID-19 vaccine distribution have been proposed. For example, Toner et al. present a “menu of options”, allowing policymakers, stakeholders, and the public to reference and select from various, potentially conflicting ethical values and their implications when deliberating vaccine allocation (Toner et al. 2020). Similarly, Wilkinson suggests surveying public opinion to inform pluralistic allocation strategies during a pandemic (Wilkinson 2023). While these approaches are not the primary focus of this paper, they complement its goals and offer valuable perspectives that could enrich future discussions.

5 It is important to note that the primary aim here is simply to show that specification is a better resolution to the compatibility problem; it does not claim that equality of opportunity is the best justified specification of equal moral concern simply because it aligns with prioritizing the worse off. Instead, I argue that its compatibility with foundational principles and coherence within the ethical framework makes it a highly defensible choice. Future work could extend this analysis to explore and compare other specifications.

6 Richardson (2000) distinguishes between a less restrictive and a more restrictive interpretation of adequate satisfaction. Under the less restrictive interpretation, principles need only be satisfied on balance, implicitly allowing for trade-offs. In contrast, the more restrictive interpretation requires that all principles be independently satisfied to a significant degree (e.g., autonomy must be respected, benefits must be substantial, and justice must be upheld). This stricter approach prioritizes categorical adherence to each principle, thereby minimizing the scope for trade-offs. This paper adopts the latter interpretation.

## References

[CR1] Aquino, Yves Saint, Wendy A. James, Jackie Leach Rogers, Farah Magrabi Scully, and Stacy M. Carter. 2021. Ethical guidance for hard decisions: A critical review of early international COVID-19 ICU triage guidelines. *Health Care Analysis: HCA: Journal of Health Philosophy and Policy*. 10.1007/s10728-021-00442-0. 10.1007/s10728-021-00442-0 PMC8547561 34704198

[CR2] Arras, John D. 2005. Rationing vaccine during an avian influenza pandemic: Why it won’t be easy. *The Yale Journal of Biology and Medicine* 78 (5): 287–300. 17132335 PMC2259161

[CR3] Bambra, Clare, Ryan Riordan, John Ford, and Fiona Matthews. 2020. The COVID-19 pandemic and health inequalities. *Journal of Epidemiology and Community Health*. 10.1136/jech-2020-214401. 10.1136/jech-2020-214401 PMC7298201 32535550

[CR4] Beall, Abigail. 2020. “The Heart-Wrenching Choice of Who Lives and Dies.” 2020. https://www.bbc.com/future/article/20200428-coronavirus-how-doctors-choose-who-lives-and-dies.

[CR5] Beauchamp, Tom L., and James F. Childress. 2013. *Principles of biomedical ethics*, 7th ed. New York: Oxford University Press.

[CR6] Beecher, Henry K. 1970. *Research and the individual: Human studies*, 1st ed. Boston: Little, Brown.

[CR7] Berlin, Isaiah. 1969. *Four essays on liberty*, 116. London, New York: Oxford Paperbacks, Oxford University Press.

[CR8] Bowleg, Lisa. 2020. We’re not all in this together: On COVID-19, intersectionality, and structural inequality. *American Journal of Public Health* 110 (7): 917–917. 10.2105/AJPH.2020.305766. 32463703 10.2105/AJPH.2020.305766 PMC7287552

[CR9] Chang, Ruth, ed. 1997. *Incommensurability, incomparability, and practical reason*. Cambridge London: Harvard University Press.

[CR10] Chang, Ruth. 2002. The possibility of parity. *Ethics* 112 (4): 659–688. 10.1086/339673.

[CR11] Cookson, Richard, and Paul Dolan. 2000. Principles of justice in health care rationing. *Journal of Medical Ethics* 26 (5): 323–329. 10.1136/jme.26.5.323. 11055033 10.1136/jme.26.5.323 PMC1733291

[CR12] Culyer, A.J., and Adam Wagstaff. 1993. Equity and equality in health and health care. *Journal of Health Economics* 12 (4): 431–457. 10.1016/0167-6296(93)90004-X. 10131755 10.1016/0167-6296(93)90004-x

[CR13] Curran, Emma J., and Stephen D. John. 2022. Must we vaccinate the most vulnerable? Efficiency, priority, and equality in the distribution of vaccines. *Journal of Applied Philosophy* 39 (4): 682–697. 10.1111/japp.12588. 10.1111/japp.12588 PMC9347581 35937030

[CR14] Dawson, Angus, David Isaacs, Melanie Jansen, Christopher Jordens, Ian Kerridge, Ulrik Kihlbom, Henry Kilham, Anne Preisz, Linda Sheahan, and George Skowronski. 2020. An ethics framework for making resource allocation decisions within clinical care: Responding to COVID-19. *Journal of Bioethical Inquiry* 17 (4): 749–755. 10.1007/s11673-020-10007-w. 32840833 10.1007/s11673-020-10007-w PMC7445717

[CR15] DIVI Deutschen Interdisziplinären Vereinigung für Intensiv- und Notfallmedizin. 2020. “Entscheidungen Über Die Zuteilung von Ressourcen in Der Notfall- Und Der Intensivmedizin Im Kontext Der COVID-19-Pandemie.” https://www.divi.de/empfehlungen/publikationen/covid-19-dokumente/covid-19-ethik-empfehlung.

[CR16] DOH Department of Health Ireland. 2020. “Ethical Framework for Decision-Making in a Pandemic.” https://www.gov.ie/pdf/?file=https://assets.gov.ie/72072/989943ddd0774e7aa1c01cc9d428b159.pdf#page=null.

[CR17] Emanuel, Ezekiel J., Govind Persad, Adam Kern, Allen Buchanan, Cécile. Fabre, Daniel Halliday, Joseph Heath, et al. 2020a. An ethical framework for global vaccine allocation. *Science* 369 (6509): 1309–1312. 10.1126/science.abe2803. 32883884 10.1126/science.abe2803 PMC8691258

[CR18] Emanuel, Ezekiel J., Govind Persad, Ross Upshur, Beatriz Thome, Michael Parker, Aaron Glickman, Cathy Zhang, Connor Boyle, Maxwell Smith, and James P. Phillips. 2020b. Fair Allocation of scarce medical resources in the time of COVID-19. *New England Journal of Medicine* 382 (21): 2049–2055. 10.1056/NEJMsb2005114. 32202722 10.1056/NEJMsb2005114

[CR19] Feiring, Eli, Reidun Førde, Søren. Holm, Ole Frithjof Norheim, Berge Solberg, Carl Tollef Solberg, and Gry Wester. 2020. *Advice on priority groups for coronavirus vaccination in Norway*. Oslo: Norwegian Institute of Public Health.

[CR20] Fitzpatrick, Meagan C., and Alison P. Galvani. 2021. Optimizing age-specific vaccination. *Science* 371 (6532): 890–891. 10.1126/science.abg2334. 33479122 10.1126/science.abg2334

[CR21] van Goozen, Sara. 2021. How should we distribute scarce medical resources in a pandemic? In *Political philosophy in a pandemic*, ed. Fay Niker and Aveek Bhattacharya, 29–41. London: Bloomsbury Publishing.

[CR22] Gupta, Rohit, and Stephanie R. Morain. 2021. Ethical allocation of future COVID-19 vaccines. *Journal of Medical Ethics* 47 (3): 137–141. 10.1136/medethics-2020-106850. 10.1136/medethics-2020-106850 33335070

[CR23] Jansen, Lynn A., and Steven Wall. 2021. Weighted lotteries and the allocation of scarce medications for Covid-19. *Hastings Center Report* 51 (1): 39–46. 10.1002/hast.1218. 33630329 10.1002/hast.1218 PMC8014103

[CR24] Jecker, Nancy S., Aaron G. Wightman, and Douglas S. Diekema. 2021. Vaccine ethics: An ethical framework for global distribution of COVID-19 vaccines. *Journal of Medical Ethics* 47 (5): 308–317. 10.1136/medethics-2020-107036. 10.1136/medethics-2020-107036 33593876

[CR25] Jöbges, Susanne, Rasita Vinay, Valerie A. Luyckx, and Nikola Biller-Andorno. 2020. Recommendations on COVID-19 triage: International comparison and ethical analysis. *Bioethics* 34 (9): 948–959. 10.1111/bioe.12805. 32975826 10.1111/bioe.12805 PMC7537413

[CR26] Joebges, Susanne, and Nikola Biller-Andorno. 2020. Ethics guidelines on COVID-19 triage—An emerging international consensus. *Critical Care* 24 (1): 201. 10.1186/s13054-020-02927-1. 32375855 10.1186/s13054-020-02927-1 PMC7202791

[CR27] Larmore, Charles E. 1987. *Patterns of moral complexity*, 1st ed. West Nyack: Cambridge University Press.

[CR28] Lawrence, Christopher, Dan J. Vick, Thomas Maryon, and Bernard J. Kerr. 2022. Ethical Allocation of COVID-19 vaccine in the united states: An evaluation of competing frameworks for the current pandemic and future events. *Journal of Public Health Policy* 43 (2): 234–250. 10.1057/s41271-022-00338-w. 35140363 10.1057/s41271-022-00338-w PMC9192923

[CR29] Lewicki, Dr Aleksandra. 2021. “Sind Menschen mit Migrationshintergrund stärker von Covid-19 betroffen?” Mediendienst Integration. https://mediendienst-integration.de/fileadmin/Dateien/MEDIENDIENST_Expertise_Covid-19_und_Migrationshintergrund.pdf.

[CR30] Liu, Yangzi, Sanjana Salwi, and Brian C. Drolet. 2020. Multivalue ethical framework for fair global allocation of a COVID-19 vaccine. *Journal of Medical Ethics* 46 (8): 499–501. 10.1136/medethics-2020-106516. 32532826 10.1136/medethics-2020-106516 PMC7316117

[CR31] März, Julian W., Anett Molnar, Søren. Holm, and Michael Schlander. 2022. The ethics of COVID-19 vaccine allocation: Don’t forget the trade-offs! *Public Health Ethics* 15 (1): 41–50. 10.1093/phe/phac001.

[CR32] Mulberry, Nicola, Paul Tupper, Erin Kirwin, Christopher McCabe, and Caroline Colijn. 2021. Vaccine rollout strategies: The case for vaccinating essential workers early. *PLOS Global Public Health* 1 (10): e0000020. 10.1371/journal.pgph.0000020. 36962089 10.1371/journal.pgph.0000020 PMC10021761

[CR33] National Academies of Sciences, Engineering, and Medicine, Board on Health Sciences Policy, Board on Population Health and Public Health Practice, Health and Medicine Division, and National Academies of Sciences, Engineering, and Medicine. 2020. *Framework for Equitable Allocation of COVID-19 Vaccine*. Edited by Helene Gayle, William Foege, Lisa Brown, and Benjamin Kahn. Washington, D.C.: National Academies Press. 10.17226/25917. 33026758

[CR34] Nuffield Council on Bioethics. 2020. “Fair and Equitable Access to COVID-19 Treatments and Vaccines.” https://www.nuffieldbioethics.org/publications/fair-and-equitable-access-to-covid-19-treatments-and-vaccines.

[CR35] Persad, Govind, Alan Wertheimer, and Ezekiel J. Emanuel. 2009. Principles for allocation of scarce medical interventions. *The Lancet* 373 (9661): 423–431. 10.1016/S0140-6736(09)60137-9. 10.1016/S0140-6736(09)60137-9 19186274

[CR36] Raus, Kasper, Eric Mortier, and Kristof Eeckloo. 2021. Ethical reflections on Covid-19 vaccines. *Acta Clinica Belgica* 77 (3): 600–605. 10.1080/17843286.2021.1925027. 34008482 10.1080/17843286.2021.1925027

[CR37] Rawls, John. 1996. Political liberalism. In *The John Dewey essays in philosophy*, 4. New York: Columbia University Press.

[CR38] Rawls, John. 1999. *A theory of justice*. Rev. Cambridge: Beecher.

[CR39] Rhodes, Rosamond. 2021. Justice in COVID-19 vaccine prioritisation: Rethinking the approach. *Journal of Medical Ethics* 47 (9): 623–631. 10.1136/medethics-2020-107117. 34108257 10.1136/medethics-2020-107117

[CR40] Richardson, Henry S. 1990. Specifying norms as a way to resolve concrete ethical problems. *Philosophy & Public Affairs* 19 (4): 279–310.

[CR41] Richardson, Henry S. 2000. Specifying, balancing, and interpreting bioethical principles. *The Journal of Medicine and Philosophy* 25 (3): 285–307. 10852336 10.1076/0360-5310(200006)25:3;1-H;FT285

[CR42] Saxena, Abha, Paul André Bouvier, Ehsan Shamsi-Gooshki, Johannes Köhler, and Lisa J. Schwartz. 2021. WHO guidance on ethics in outbreaks and the COVID-19 pandemic: A critical appraisal. *Journal of Medical Ethics* 47 (6): 367–373. 10.1136/medethics-2020-106959. 10.1136/medethics-2020-106959 33789948

[CR43] Scheunemann, Leslie P., and Douglas B. White. 2011. The ethics and reality of rationing in medicine. *Chest* 140 (6): 1625–1632. 10.1378/chest.11-0622. 22147821 10.1378/chest.11-0622 PMC3415127

[CR44] Swiss Academy of Medical Sciences. 2020. “COVID-19 Pandemic: Triage for Intensive-Care Treatment under Resource Scarcity.” https://www.sams.ch/en/Ethics/Topics-A-to-Z/Intensive-care-medicine/Triage-intensive-care-medicine.html. 10.4414/smw.2020.20229 32208495

[CR45] Toner, Eric, Anne Barnill, Carleigh Krubiner, and Justin Bernstein. 2020. *Interim Framework for COVID-19 Vaccine Allocation and Distribution in the United States*. Baltimore: The Johns Hopkins Center for Health Security.

[CR46] Wasserman, David, Govind Persad, and Joseph Millum. 2020. Setting priorities fairly in response to COVID-19: Identifying overlapping consensus and reasonable disagreement. *Journal of Law and the Biosciences* 7 (1): 1–12. 10.1093/jlb/lsaa044. 10.1093/jlb/lsaa044 PMC7337850 32879733

[CR47] White, Douglas B., Mitchell H. Katz, John M. Luce, and Bernard Lo. 2009. Who should receive life support during a public health emergency? Using ethical principles to improve allocation decisions. *Annals of Internal Medicine* 150 (2): 132–138. 19153413 10.7326/0003-4819-150-2-200901200-00011 PMC2629638

[CR48] White, Douglas B., Erin K. McCreary, Chung-Chou H. Chang, J. Mark Schmidhofer, Ryan Bariola, Naudia N. Jonassaint, Govind Persad, et al. 2022. A multicenter weighted lottery to equitably allocate scarce COVID-19 therapeutics. *American Journal of Respiratory and Critical Care Medicine* 206 (4): 503–506. 10.1164/rccm.202201-0133LE. 35549997 10.1164/rccm.202201-0133LE

[CR49] Wilkinson, Dominic. 2023. Pluralism and allocation of limited resources: Vaccines and ventilators. In *Pandemic ethics: From COVID-19 to disease X*, ed. Julian Savulescu and Dominic Wilkinson. Oxford: Oxford University Press. 37343115

[CR50] Williams, Bernard. 1981. *Moral luck*. Cambridge: Cambridge University Press.

[CR51] Williams, Jane H., and Angus Dawson. 2020. Prioritising access to pandemic influenza vaccine: A review of the ethics literature. *BMC Medical Ethics* 21 (1): 40. 32408869 10.1186/s12910-020-00477-3 PMC7224123

[CR52] Wolff, Jonathan, and A. De-Shalit. 2007. *Disadvantage*. Oxford: Oxford University Press.

[CR53] Wolff, Jonathan, and A. De-Shalit. 2021. Risk, disadvantage and the COVID-19 crisis. In *Political philosophy in a pandemic*, ed. Fay Nicker and Aveek Bhattacharya, 13–28. London: Bloomsbury Publishing.

[CR54] World Health Organization. 2020. “WHO Concept for Fair Access and Equitable Allocation of COVID-19 Health Products.” https://www.who.int/docs/default-source/coronaviruse/who-covid19-vaccine-allocation-final-working-version-9sept.pdf.

